# Preparation of a Polyaniline-Modified Hybrid Graphene Aerogel-Like Nanocomposite for Efficient Adsorption of Heavy Metal Ions from Aquatic Media

**DOI:** 10.3390/polym15051101

**Published:** 2023-02-22

**Authors:** Tatiana S. Kuznetsova, Alexander E. Burakov, Irina V. Burakova, Tatiana V. Pasko, Tatiana P. Dyachkova, Elina S. Mkrtchyan, Anastasia E. Memetova, Oksana A. Ananyeva, Gulnara N. Shigabaeva, Evgeny V. Galunin

**Affiliations:** 1 Department of Technology and Methods of Nanoproducts Manufacturing, Tambov State Technical University, 106/5, Building 2, Sovetskaya St., 392000 Tambov, Russia; 2 Department of Organic and Ecological Chemistry, Institute of Chemistry, University of Tyumen, 6 Volodarskogo St., 625003 Tyumen, Russia

**Keywords:** carbon nanotubes, graphene oxide, polymer, polyaniline, aerogel, modification, carbonization, lead ions, adsorption, kinetics

## Abstract

This paper considers the synthesis of a novel nanocomposite based on reduced graphene oxide and oxidized carbon nanotubes modified with polyaniline and phenol-formaldehyde resin and developed through the carbonization of a pristine aerogel. It was tested as an efficient adsorbent to purify aquatic media from toxic Pb(II). Diagnostic assessment of the samples was carried out through X-ray diffractometry, Raman spectroscopy, thermogravimetry, scanning and transmission electron microscopy, and infrared spectroscopy. The carbonized aerogel was found to preserve the carbon framework structure. The sample porosity was estimated through nitrogen adsorption at 77 K. It was found that the carbonized aerogel predominantly represented a mesoporous material having a specific surface area of 315 m^2^/g. After carbonization, an increase in smaller micropores occurred. According to the electron images, the highly porous structure of the carbonized composite was preserved. The adsorption capacity of the carbonized material was studied for liquid-phase Pb(II) extraction in static mode. The experiment results showed that the maximum Pb(II) adsorption capacity of the carbonized aerogel was 185 mg/g (at pH 6.0). The results of the desorption studies showed a very low desorption rate (0.3%) at pH 6.5 and a rate of about 40% in a strongly acidic medium.

## 1. Introduction

The contamination of water resources with toxic, inorganic elements appears to be one of the largest global environmental issues. The presence of such species in aquatic media is caused by both natural and anthropogenic sources [1,2]. It is known that heavy metals are not biodegradable, can accumulate in groundwater, can be transmitted through the food chain, and accordingly, can accumulate in the body, posing a huge threat to the ecosystem and human health [3]. They usually enter natural waters through wastewater coming from the following sources: heavy and chemical industries [1].

Among such species, lead ions (Pb(II)) are considered one of the most toxic; their excessive accumulation can bring severe damages to the nervous system and internal organs, result in the development of cancer and even in death [3,4]. Therefore, the task of purifying wastewater from this contaminant is quite relevant.

There exist several comprehensive methods for the disposal of toxic metals from aquatic media, such as ion exchange, adsorption, filtration, and chemical precipitation. Among them, adsorption is considered the most popular technique due to low production cost, high removal rate, and process efficiency [2]. 

At present, the uses of many conventional sorbents for adsorption have several disadvantages; therefore, aerogels based on carbon nanomaterials, such as carbon nanotubes (CNTs) and graphene oxide (GO), are being actively studied. These materials possess a large surface area, high porosity, low density, good conductivity, and fire resistance, as well as chemical, thermal, and mechanical stability [5]. 

Specific surface area and pore size affect the adsorption capacity of materials. Therefore, an urgent task is to obtain highly porous materials with developed surfaces. Modern scientific approaches involve the use of freezing or supercritical drying to form a porous framework. Evaporative drying is considered texture-destroying due to capillary forces acting on the pores during drying. Consequently, organic gels are usually dried through supercritical drying [6]: after changing the solvent (water for acetone or ethanol, and then for CO_2_), gels are dried under supercritical conditions. Thus, surface tension is eliminated and shrinkage does not occur, and the dried materials, called “aerogels”, preserve most of the original gel texture [6].

For instance, in a study [7], a GO-based aerogel was synthesized through a unidirectional sublimation drying method. The study showed that the adsorption kinetics corresponded to a pseudo-second-order model, with active adsorption of lead ions during the first 90 min, whereas the adsorption capacity reached 158.7 mg/g. 

The authors of another study [8] synthesized an aerogel based on alginate-crosslinked GO. It was produced from composite materials via a sol-gel method followed by sublimation drying. This material demonstrated selectivity for Pb(II), which was compared to other metals having the same valence and was about ten times more efficient than standard activated carbon. Adsorption studies showed that this novel material possessed a high adsorption capacity determined within 6 h at 22 °C and pH 5.0. Moreover, it could be regenerated by washing with some acids. 

In another study [9], a graphene aerogel with high mechanical strength, as well as controlled density and pore volume, was obtained through soaking a graphene hydrogel in an ammonia solution. In this material, the density and pore volume were controlled by adjusting the ammonia solution concentration. The material was found to possess a Pb(II) adsorption capacity of 80 mg/g, and it could be easily separated from water after the adsorption process. Adsorption studies were carried out at room temperature (22 °C).

In a previous study [10], a three-dimensional porous GO-based xerogel was developed through molecular self-assembly of the GO on a chitosan matrix, and its application was studied for the removal of various heavy metal ions from wastewater. Adsorption studies demonstrated that this material had a Pb(II) adsorption capacity of 40.92 mg/g at 25 °C and a solution at pH 9.0. Kinetic studies showed that a sharp decrease in the metal ion content in the solution occurred within 8 h, but the material was completely saturated within 24 h.

Carbonization is used for postprocessing xerogels, cryogels, and aerogels to obtain hybrid materials based on carbon 3D structures. The process is performed in an inert atmosphere (usually nitrogen or argon) or in a vacuum at high temperatures. This removes residual water and volatile organic components. Carrying out carbonization at a high temperature promotes the formation of graphene-like structures. The authors of [11] reduced GO with ascorbic acid and annealed it at 500 °C. Postprocessing was used to remove residual oxygen-containing groups from graphene aerogels. The resulting material possessed supercompressibility, superelasticity, and superfatigue resistance compared to material that did not go through the annealing step.

Quite often, to improve the properties of aerogels, they are modified with polyaniline (PANI), a conductive polymer. In combination with nanomaterials, PANI demonstrates not only improved mechanical characteristics, but also increased productivity [12]. Thus, studies aimed at improving the adsorption efficiency of PANI, the ergonomics of its use, and its general applicability are considered topical. Although PANI is highly efficient in removing heavy metals, the particles easily aggregate during chemical synthesis, thereby limiting its adsorption capacity and practical application [13,14]. 

A particular property of PANI lying in the fact that it can serve as a carbon precursor for manufacturing nitrogen-doped carbon materials is of interest to researchers. The controlled carbonization of polymers is one of the most important polymer reactions [15]. Since the morphology of PANI is largely preserved during carbonization, its nanostructures often serve as precursors for obtaining controlled morphology structures. Usually, PANI carbonization is carried out in an inert, reducing atmosphere or vacuum at a constant temperature of 300–1200 °C for times ranging from several minutes to several hours [16].

As an example, a magnetic nanocomposite consisting of PANI and multi-walled carbon nanotubes (PANI/MWCNTs) described in [17] can be mentioned. This material showed a Pb(II) adsorption capacity of 22.2 mg/g within 24 h. The studies were carried out at room temperature (20 °C) with an initial solution pH of 5.0. The PANI/MWCNTs magnetic composite could be separated and recovered from the aqueous solution by magnetic separation. 

Another study [18] considered a procedure for synthesizing a ternary nanocomposite consisting of lignosulfonate, GO, and PANI. Its maximum adsorption capacity was found to be 216.4 mg/g at 30 °C and pH 5.0. However, the contact time was about 4 h at a recovery rate of 98.3%. The synergistic effect of functional groups on this composite played a key role in the adsorption. 

Thus, the authors of the present study aim at developing an aerogel-like nanostructured composite based on graphene structures modified with a target polymer agent possessing a pronounced nitrogen functionality to efficiently remove one of the most dangerous and toxic inorganic pollutants (lead) from aquatic systems.

## 2. Materials and Methods

### 2.1. Reagents

The following original reagents were used to obtain the nanocomposite: reduced GO (rGO, synthesized as an aqueous paste containing 2.42 wt.% dry matter by the reduction of GO with ascorbic acid) produced by NanoTechCenter LLC, Tambov, Russia; oxidized carbon nanotubes (oCNTs, synthesized as an aqueous paste containing 11.48 wt.% dry matter by the oxidation of Taunit-M CNTs with a sodium hypochlorite solution) also manufactured by NanoTechCenter LLC; PANI aqueous paste (containing 11% dry matter—PANI-base); distilled (demineralized) water (according to Russian National Standard (GOST) R 58144-2018); and resole water-soluble phenol-formaldehyde resin (PFR, “Fenotam-GR-326” obtained from Krata PJSC Industrial Group, Tambov, Russia.

### 2.2. Laboratory Method for Obtaining a Hydrogel

An initial hydrogel was synthesized at room temperature without pretreating the materials. The original components—PANI aqueous paste, oCNTs, rGO, and resole water-soluble PFR—were mixed in a dry matter ratio of 1:1:1:1 in distilled water, bringing the total mass to 100 g.

Next, the resulting mixture was processed with an IL-10 ultrasonic unit (Ultrasonic Technology, INLAB CJSC, St. Petersburg, Russia) for 1 h at a frequency of 22 kHz to destroy particle aggregates and uniformly distribute the solution components. Then, 25 mL of 9% acetic acid was added to the resulting mixture and kept for 1 h to coagulate the solution. After that, the mixture was filtered with a microfilter and washed with distilled water to remove reaction byproducts. Excess moisture was removed over several minutes using a vacuum filter.

### 2.3. Laboratory Method for Obtaining the Nanocomposite Material (Aerogel)

For the subsequent production of a pristine aerogel, in the original hydrogel, the water was replaced with isopropyl alcohol, and the mixture was dried under supercritical conditions (at 235.3 °C and 47.6 atm) in a high-pressure reactor (Nano-Mag Technologies Pvt. Ltd., Mumbai, India).

Next, the aerogel was annealed at 800 °C in an inert gas environment for 1 h in a tube furnace to carbonize the final material (carbonized aerogel).

**Figure 1:**
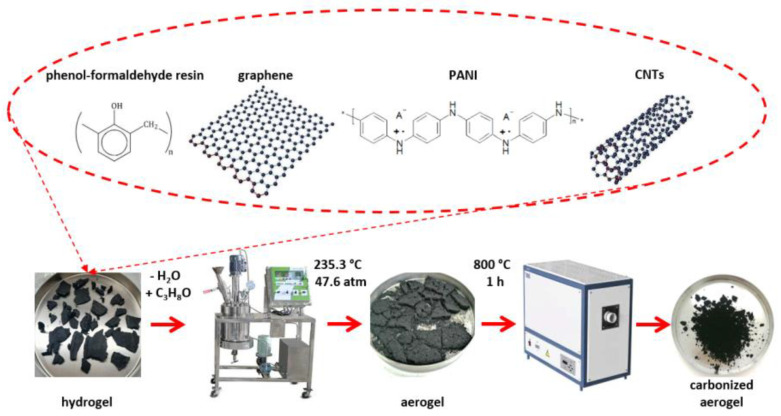
A scheme for obtaining the carbonized material.

Figure 1 shows the scheme for obtaining the carbonized aerogel.

### 2.4. Material Characterization

The specific surface area, volume, and pore size of the carbonized aerogel were studied using nitrogen adsorption at 77 K with an Autosorb iQ automatic analyzer (Quantachrome Instruments, Boynton Beach, FL, USA). The structural characteristics were studied using X-ray phase analysis with a Thermo Scientific ARL Equinox 1000 X-ray diffractometer (TechTrend Science Co., Ltd., Tainan City, Taiwan). A MERLIN electron microscope (Carl Zeiss, Jena, Germany) and a JEM-2010 instrument (JEOL Ltd., Tokyo, Japan) were used for scanning and transmission electron microscopy (SEM and TEM) investigations, respectively, to study surface topography and morphology. The chemical analysis of the material composition and structure was performed through Raman spectroscopy with a DXR Raman microscope (Thermo Fisher Scientific, Waltham, MA, USA). Thermogravimetry (TG) and differential scanning calorimetry (DSC) analyses were performed with a NETZSCH STA 449 F3 Jupiter simultaneous thermal analysis instrument (NETZSCH-Feinmahltechnik GmbH, Selb, Germany).

### 2.5. pH Effect Studies

To determine the pH effect on the adsorption characteristics of the synthesized nanocomposites, model solutions were prepared in acetic-acetate buffer systems at pH levels ranging from 2.0 to 9.0. The pH value was measured using a HI 2210 benchtop pH meter with an automatic calibration function (HANNA Instruments, Woonsocket, Rhode Island, USA). To perform this, required amounts of acetic acid and sodium hydroxide solutions (for pH 4.0, 6.0, and 9.0), as well as hydrochloric and amino-acetic acid solutions (for pH 2.0), were added to deionized water. An amount of 0.005 g of the adsorbent was placed into 50 mL conical test tubes with 30 mL of Pb(II) nitrate solution (initial concentration *C*_0_ = 100 mg/L). The absorption time was 30 min. After the solution was filtered, the solid phase was separated, and the equilibrium Pb(II) concentration was determined through energy-dispersive X-ray fluorescence spectrometry using a Thermo Scientific ARL Quant’X instrument (Thermo Fisher Scientific). The sensitivity of the spectrometer was in the range from <1 ppm to 100%, and the measurement time of one element was from 10 to 60 s. The adsorption capacity value (*q_t_*, mg/g) was determined according to the following formula:

(1) $$q_{t}=\frac{V\left(C_{0}-C_{e}\right)}{m},$$

where *C*_0_ and *C_e_* are the initial and final (equilibrium) concentrations of the metal ions in the solution in mg/L, respectively; *V* is the volume of the solution in L; and *m* is the weight of the adsorbent in g. 

### 2.6. Kinetic Studies on the Carbonized Aerogel

The adsorption capacity of the synthesized carbonized aerogel was determined as follows. An amount of 0.005 g of adsorbent was placed into test tubes with 30 mL of a Pb(II) solution (initial concentration *C*_0_ = 100 mg/L) in a buffer solution (pH 6.0) at room temperature (21 °C). Then, the tubes were end-over-end shaken with a Multi Bio RS-24 programmable rotator (Biosan, Riga, Latvia) at a rotation speed of 120 rpm for 10, 20, 30, 40, and 60 min. After that, the solution was filtered to separate the solid phase, and the final concentration of lead ions was measured using energy-dispersive X-ray fluorescence spectrometry with a Thermo Scientific ARL Quant’X instrument (Thermo Fisher Scientific).

### 2.7. Desorption Studies on the Carbonized Aerogel

To determine the desorption value, adsorbent samples were taken after completion of the adsorption cycle. Each sample containing the final (equilibrium) Pb(II) concentration was placed into a polyethylene tube containing 30 mL of distilled water adjusted to pH 6.5, thereby simulating a desorption process under normal conditions, and then shaken for 60 min with periodic sampling at indicated time intervals.

To assess the desorption rate after exposing the adsorbent to a more aggressive medium, a 2% HCl solution potentially allowing complete desorption to be achieved was used. To perform this, after the adsorption process, a weighed portion of the adsorbent was placed into a polyethylene test tube containing 30 mL of HCl solution and was then also shaken for 60 min with periodic sampling to assess the concentration of Pb(II) released into the solution.

## 3. Results and Discussion

### 3.1. Physical-Chemical Properties of the Nanocomposites

**Figure 2:**
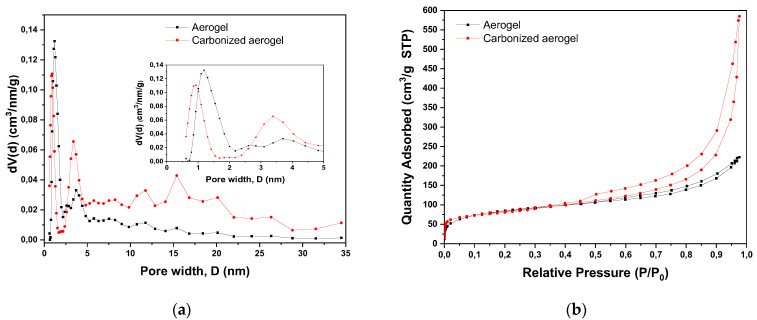
Pore size distribution (**a**) and N_2_ adsorption–desorption isotherms (**b**) obtained for the nanocomposite before and after carbonization (pristine and carbonized aerogel, respectively).

The pore size distribution and N_2_ adsorption–desorption isotherms at 77 K for the noncarbonized and carbonized samples are shown in Figure 2 and Table 1, respectively.

The pore size distribution plot (Figure 2a) demonstrates increases in the numbers of micropores and mesopores after carbonization. In this case, the volume of micropores (*V_micro_*) calculated by the DFT method assuming slit-like pores (QSDFT model) significantly increased for the carbonized aerogel compared to the pristine (noncarbonized) material.

Figure 2b shows a solid-state isotherm (type-IV) typical of porous adsorbents in which the adsorption process was limited by the micropore volume [19]. The H3-type hysteresis loop for the carbonized aerogel and the H4-type hysteresis loop for the pristine aerogel did not exhibit limiting adsorption at high relative pressures (*P/P*_0_). It is generally accepted that this type of isotherm corresponds to aggregates of lamellar particles, leading to slit-like pores. The type of hysteresis loop corresponded to micro-meso-porous carbons, and the small area with a bulge in the initial section of the isotherm may indicate a small amount of micropores.

According to Figure 2a, the aerogel carbonization resulted in a shift in the pore distribution maximum from 1.3 nm (aerogel) to 0.9 nm (carbonized aerogel) for the micropores. The mesopore distribution was represented by wider areas with maxima around 3.8, 14, 15, and 20 nm for both the composites. However, as shown in Table 1, the mesopore volume doubled from 0.258 to 0.535 cm^3^/g after carbonization. This fact may presumably be explained by the burning out of thin walls between neighboring aerogel micropores represented, for instance, by unstructured carbon or the rGO/oCNT combination, which ultimately led to the formation of a larger number of mesopores and new micropores.

**Figure 3:**
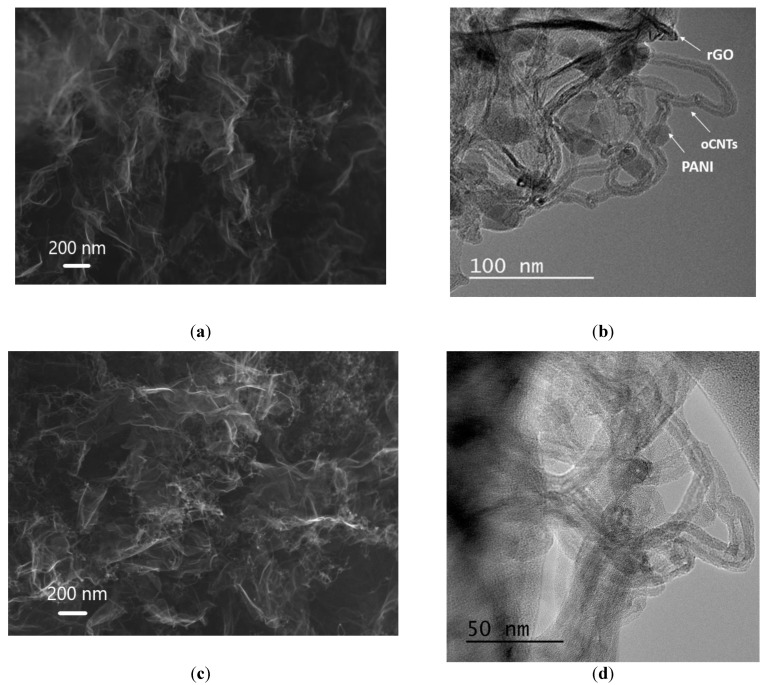
SEM (100.00 kx magnification) and TEM images of the pristine aerogel (**a**,**b**) and carbonized aerogel (**c**,**d**), respectively.

Figure 3 demonstrates the SEM (Figure 3a,c) and TEM (Figure 3b,d) images of the structures of the pristine and carbonized aerogels, respectively. The presented SEM images of the materials allow for concluding that the concept herein proposed to develop a composite in which the oCNTs acted as a structure-forming agent and a supporting framework that prevented rGO sheet aggregation was generally confirmed. In the final composite, both the rGO and CNTs, in addition to their structure-forming role, appeared to be carriers of a large number of active adsorption sites and functional groups. Thus, the resulting samples had highly porous structures. The oCNTs were located on the surface of the rGO sheets, whereas the PANI particles preserve their shape during carbonization. There was an increase in the inhomogeneous layer located on the surface of the 3D structures.

**Figure 4:**
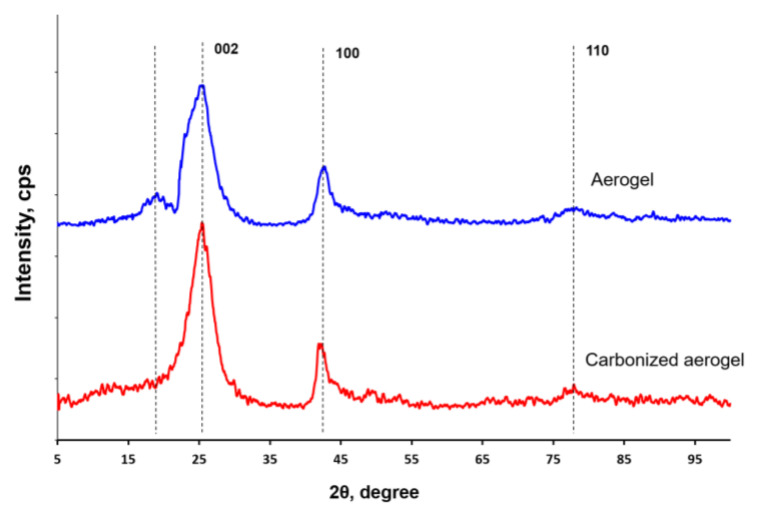
X-ray diffraction patterns of the pristine and carbonized aerogels.

Figure 4 shows the X-ray diffraction patterns of the synthesized nanocomposites. For the pristine aerogel, four peaks could be observed at 2θ = 19.11, 25.49, 42.61, and 78.44°, whereas for the carbonized aerogel, only three peaks were visible at 2θ = 25.38, 42.32, and 78.26°. From these diffraction patterns, it can be assumed that the noncarbonized composite containing the rGO, oCNTs, and PANI represented a material possessing a layered structure with an interlayer distance close to that of graphene layers in graphite. At the same time, in the composite after carbonization, no peak at ~19° indicating ordered PANI structures before carbonization was observed, but peaks at ~25, 42, and 78° characteristic of carbon nanostructures were preserved.

Table 2 presents the interplanar distance (d) values estimated for the pristine and carbonized aerogels. As can be seen from this table, during carbonization in the material, an ordered structure of the carbon framework was preserved.

**Figure 5:**
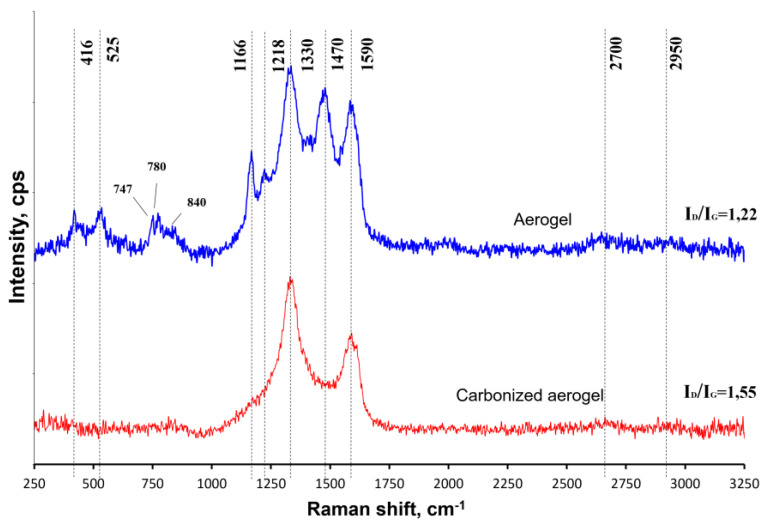
Raman spectra recorded for the pristine and carbonized aerogels.

Figure 5 shows the Raman spectra recorded for the pristine and carbonized aerogels. The spectra of the carbonized sample were represented by two characteristic modes: *G* (1500–1600 cm^−1^), which was due to the vibrations of sp^2^-hybridized carbon atoms in the graphene layer plane within the lattice, and *D* (1250–1450 cm^−1^), which was associated with the presence of sp^3^-hybridized carbon atoms that appear when topological defects take place in graphene layers or are related to the availability of amorphous carbon particles. The peaks at 2700 and 2900 cm^−1^ were hardly noticeable [20].

The changes in the peak intensity ratios in the pristine and carbonized aerogels can be explained by the transformation of the PANI. This was due to its conversion into a weakly structured form. 

In addition, due to the changes in the PANI and PFR and the transformation of the oxygen-containing groups, an increase in the number of sp^3^-hybridized atoms (reflected by the *D* peak on the spectra) took place, which accordingly led to an increase in the *I_D_/I_G_* ratio for the sample after carbonization from 1.22 to 1.55 [21].

**Figure 6:**
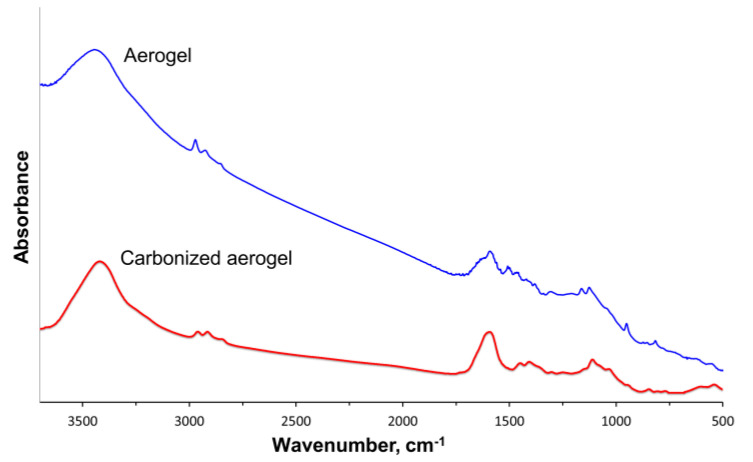
IR spectra recorded for the pristine and carbonized aerogels.

The infrared (IR) spectra recorded for the pristine and carbonized aerogels are presented in Figure 6.

The IR spectra of the pristine aerogel contained a broad absorption band in the region above 3000 cm^−1^, which was associated with the availability of the hydroxyl groups or adsorbed water. The peaks at 2970, 2930, and 2850 cm^−1^ were due to vibrations of the *C-H* bonds in the alkyl groups that may be present on the rGO surface. Due to the availability of the PANI in the pristine material, the spectra exhibited peaks at 1590, 1506, and 1460 cm^−1^, which, according to [22], corresponded to quinone ring (1590 and 1506 cm^−1^) and benzene ring (1460 cm^−1^) stretching deformations. The peaks at 1161 and 1129 cm^−1^ appeared due to the presence of the N-H bonds in the amino groups.

As can also be seen from Figure 6, for the carbonized aerogel, the peak intensity decreased compared to the pristine sample. The spectrum of the carbonized material had a peak in the range of 3200–3700 cm^−1^ corresponding to the stretching vibrations of the −*OH* bond, and the intensity of the peaks in the region of 2800–3000 cm^−1^ significantly decreased. In the region of 1700–500 cm^−1^, a peak corresponding to the *C=C* bond could be observed, together with two halos in the regions of 1500–1350 and 1350–900 cm^−1^ [23,24,25,26]. This change could be associated with the behavior of the composite components under the influence of high temperatures. 

The PANI macromolecules started to degrade and most of the oxygen- and nitrogen-containing groups were removed, resulting in the formation of a carbonized material with a highly developed surface.

**Figure 7:**
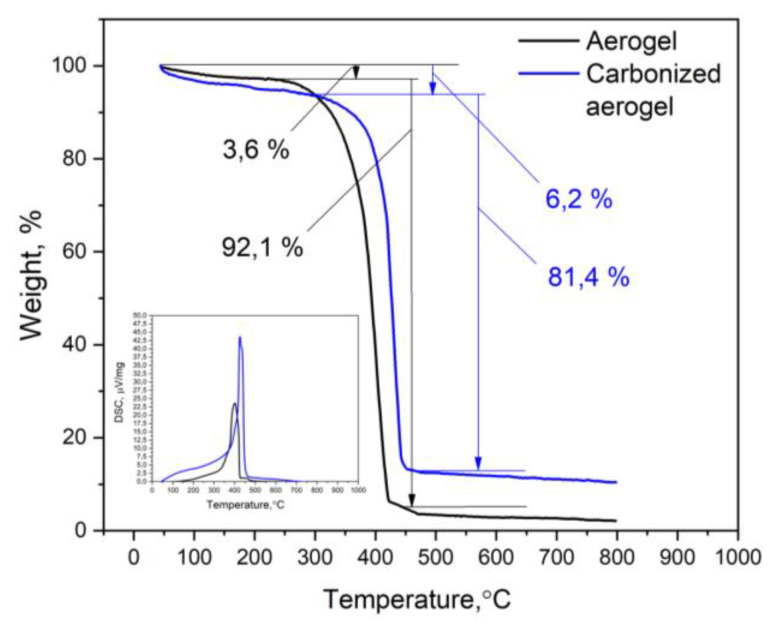
TG and DSC analyses of the pristine and carbonized aerogels.

Thermal stability is an important operational characteristic of adsorbents. The TG analysis was carried out at a heating rate of 10 °C/min in air up to 800 °C, as shown in Figure 7. When the aerogel sample was heated within the temperature range of 40–150 °C, water and light molecular compounds evaporated, thereby leading to a weight loss of 3.6%. At higher temperatures (250–400 °C), a gradual loss of weight and a residual weight of about 3–5% at 800°C could be observed. Decomposition occurred with heat release; the maximum heat release process occurred at ~400 °C. 

The thermal decomposition natures of the carbonized materials were similar. The TG curve (Figure 7) showed a residual weight of 10–12% at 800 °C and a shift in peak weight loss at higher temperatures (about 350–450 °C). According to the DSC curve, the maximum heat release occurred at 440 °C. In addition, the decrease in mass loss at the final step (450–800 °C) for the carbonized aerogel could be associated with lower reactivity and a decrease in the content of volatile substances. The latter region, also known as the passive pyrolysis region, which ranges starting from temperatures above 600 °C, corresponded to the degradation of complex organic compounds [27]. 

In addition to the shift in the position of the maximum on the DSC curve, as a result of material carbonization from 400 to 440 °C, a significant change in the height and area of the corresponding peak could be observed. The oxidative degradation of the carbonized material was accompanied by a much higher thermal effect than that of the pristine aerogel. This was due to the fact that the carbonized aerogel containing a noticeably smaller number of functional groups is characterized by a less-defective structure. According to [28], this leads to an increase in heat release during material combustion.

### 3.2. Optimal pH Range

**Figure 8:**
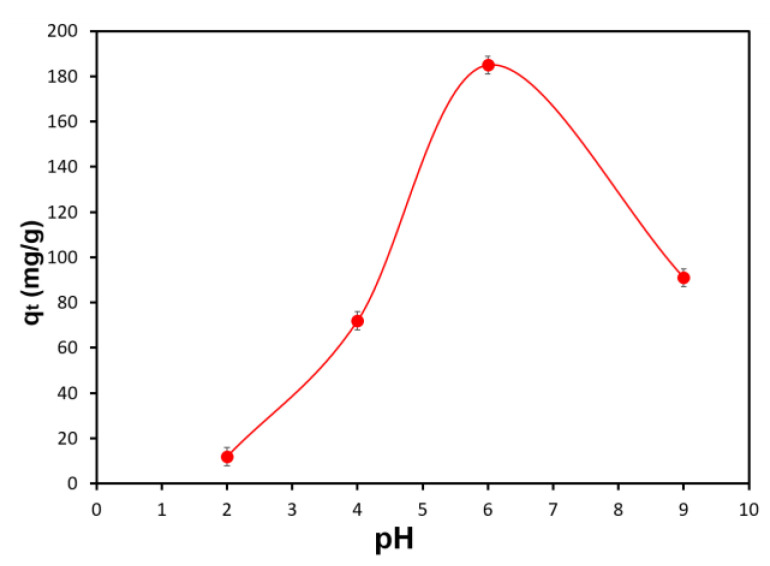
The pH effect on the Pb(II) adsorption capacity of the carbonized aerogel.

Figure 8 demonstrates the pH effect on the Pb(II) adsorption capacity of the carbonized aerogel.

When working with solutions of heavy metal ions, it is required to consider their form in aquatic media and the pH value corresponding to the onset of the precipitation of metal hydroxides. Depending on pH value, lead in aqueous solutions takes the following forms: at pH < 6.0—Pb(II) ions; at pH 7.0–11.0—Pb(II) ions, basic hydroxide Pb(OH)^+^, amphoteric hydroxides, Pb(OH)_2_, and Pb(OH)^3-^; and at pH > 11—amphoteric hydroxide Pb(OH)^3-^. The beginning of the precipitation of lead hydroxide corresponds to pH 6.4 (at 1 M). Depending on solution concentration, the precipitation pH ranges from 6.4 to 9.0 [29].

Relatively low absorption rates in acidic media at pH~2.0–4.0 are associated with a high content of competing hydronium ions [30,31]. Within this range, the predominant form is ionic (Pb(II)). During adsorption, there was competition between Pb(II) and hydrogen ions on the adsorption sites of the adsorbent. In the pH range from 4.0 to 6.0, the adsorption of metal ions increased, with a maximum at pH 6.0. In this region, the concentration of hydrogen ions decreased. A significant increase in the Pb(II) adsorption capacity of the synthesized composite material could be observed at pH 6.0. With further increase in the solution pH, the Pb(II) adsorption capacity of the material decreased, which was associated with the precipitation of the metal hydroxides (pH~7.0–10.0). In this case, in the solution phase, the metal was almost completely available in the precipitated form and did not actually interact with the active surface of the adsorbent. Based on the results of the experiment, further studies were carried out in buffer aqueous solutions at pH 6.0.

### 3.3. Kinetic Studies on the Adsorption Capacity of the Carbonized Aerogel

Studying adsorption kinetics and diffusion mechanisms is important for understanding the adsorption rate, which is required to determine the efficiency of the material being tested, elucidating the process mechanism. 

**Figure 9:**
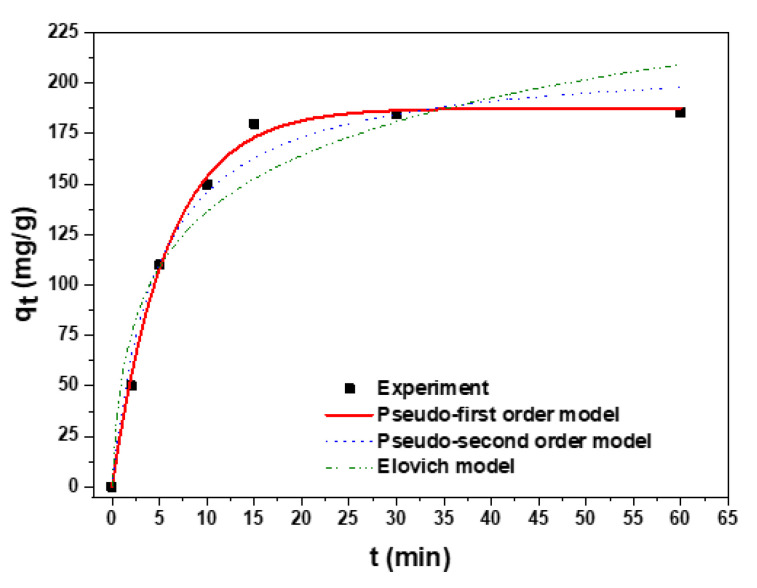
Graphical representation of the experimental data fitted to pseudo-first-order, pseudo-second-order, and Elovich kinetic models describing Pb(II) adsorption on the carbonized aerogel.

As shown in Figure 9, the rate of the Pb(II) adsorption on the carbonized aerogel was very high, which is one of the main advantages of this material over similar composites known from international publications. From this figure, it can be seen that the Pb(II) ions were significantly removed in just 5 min, and after 15 min, no noticeable changes in the results on the target pollutant removal was observed, meaning that equilibrium was reached.

The kinetics of the Pb(II) adsorption on the carbonized sample were analyzed using pseudo-first-order [32], pseudo-second-order [33], and Elovich [34] equations, whereas the diffusion mechanisms were adjusted to the Weber–Morris [35] and Boyd [36] models (Table 3, Figure 8).

To establish which of these models most qualitatively described the Pb(II) adsorption on the carbonized aerogel, their correlation coefficients *R^2^* (Table 4) were compared.

The nonlinear form curves of the models are shown in Figure 8. In addition, the corresponding kinetic and diffusion parameters are presented in Table 4. The results demonstrate that the pseudo-first-order equation had the highest correlation coefficient (R^2^ of 0.9972). To determine the energetic inhomogeneity of the adsorbent surface, the experimental data were processed using the Elovich model. The R^2^ value for this model was 0.9331, assuming that the energetic inhomogeneity of the aerogel surface had little effect on the overall rate of the adsorption process [34].

**Figure 10:**
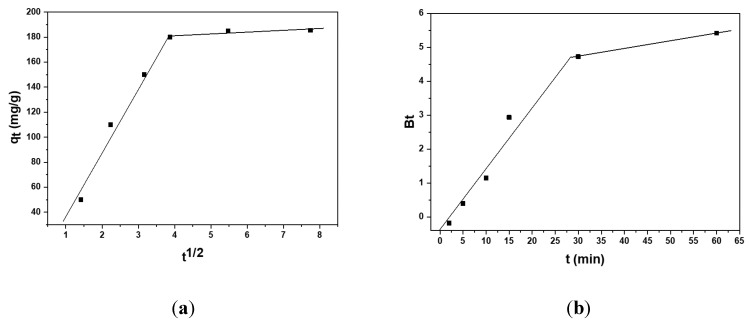
The Weber–Morris (**a**) and Boyd (**b**) diffusion models constructed for the Pb(II) adsorption on the carbonized aerogel.

To gain insight into the rate-controlling step of the entire adsorption process, the kinetic data were also fitted to the Weber–Morris (intraparticle diffusion) and Boyd (external, or film) diffusion models. The plots of these models are shown in Figure 10, and the calculated parameters are also given in Table 4.

As a rule, the adsorption process goes through several steps, resulting in different segments that appear on a Weber–Morris plot. As shown in Figure 10a, the q_t_ vs. t^1/2^ plot had two linear sections: (1) the intraparticle diffusion step and (2) the equilibrium step. Moreover, the first linear segment did not pass through the origin, indicating that intraparticle diffusion was not the only factor limiting the process rate. To confirm the above results, the Boyd (external, or film, diffusion) model was also studied. The plot presented in Figure 10b was linear at the beginning of the adsorption, and it passed through the origin, indicating that film diffusion was the rate controlling step at this period. Thus, both the diffusion of the target ions through the solution film and the diffusion in the adsorbent grain contributed to the overall rate of the process; therefore, the Pb(II) adsorption on the carbonized aerogel proceeded in a mixed diffusion model.

Thus, the conclusions drawn from the analysis of the chemical kinetics and diffusion models correlated with the results obtained from estimating the parameters of the nanocomposite porous structure through BET modeling using nitrogen adsorption. It should be noted once again that the applicability of the Elovich model was low, thereby indicating the homogeneity of the nanocomposite surface. Therefore, the use of the BET multimolecular adsorption model was justified, since it is valid for adsorption on a homogeneous surface when the energy of adsorbate–adsorbent interactions is the same at all the surface regions [37]. The results obtained during the processing of the kinetic experimental data are in good agreement with the BET theory. According to the latter, if adsorbate–adsorbent interactions prevail in a system (i.e., correspondence to the pseudo-first order model), then at first, a practically filled monolayer is formed on the adsorbent surface, followed by subsequent layers. This fact was confirmed by the S-shaped nitrogen adsorption isotherm (Figure 2b) [19]. The increased steepness of the curve indicated not only an increase in the number of micropores, but also a significant contribution of transport mesopores, the volume of which also increased significantly as a result of the carbonization, which considerably activated the diffusion of the adsorbate into the material structure and its accumulation in the developed porous space formed by a large number of micropores.

Studies related to the extraction of heavy metals from aqueous solutions are of scientific interest to many researchers around the world. Table 5 presents a comparative analysis of the adsorption capacities of the most widely used adsorbents [38,39,40,41,42,43,44,45,46,47]. The above information shows that the carbonized nanocomposite aerogel developed by the authors of the present work is a very competitive material since its use makes it possible to achieve high adsorption capacity values with smaller adsorbent weights in a shorter period of time.

### 3.4. Desorption Tests

**Figure 11:**
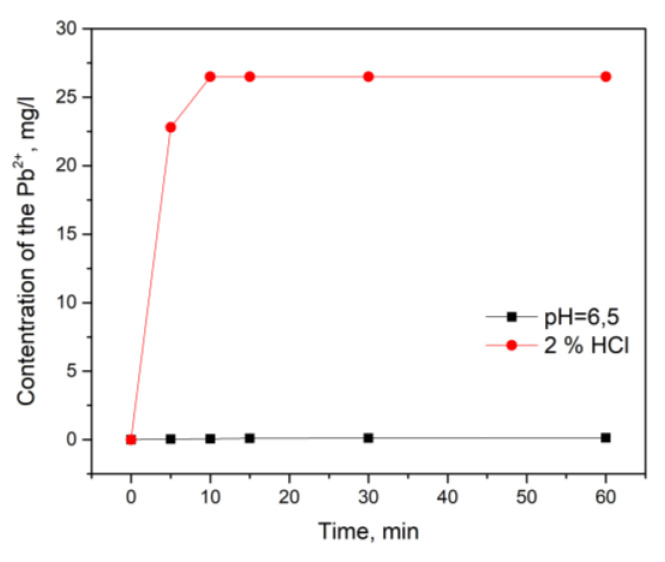
Pb(II) desorption from the carbonized aerogel at neutral and strongly acidic pH values. The equilibrium Pb(II) concentration in the solutions before desorption was ~69.2 mg/L.

The dependences presented in Figure 11 make it possible to assert that the desorption rate value was extremely small (~0.3% of the pollutant adsorbed) under normal conditions in an aqueous medium having a pH value close to neutral.

However, when exposed to a strong acid, the desorption was found to be much more active and eventually reached a value close to 40% of the pollutant adsorbed. At the same time, it can be argued that, under these conditions, complete displacement of Pb(II) into the surrounding solution did not occur.

## 4. Conclusions

The authors developed a method for synthesizing a nanocomposite material based on rGO and oCNTs modified with PANI and PFR. To form a porous carbon structure, postprocessing (carbonization) of the material was employed, which made it possible to significantly improve the characteristics of the porous space compared to the pristine aerogel. The BET specific surface area was found to increase from 289 to 315 m^2^/g after carbonization. The micropore and mesopore volumes increased 3.5 and 2.0 times, respectively. Aerogel carbonization resulted in a shift in the pore distribution maximum from 1.3 nm (pristine aerogel) to 0.9 nm (carbonized aerogel) for the micropores. 

The physical and chemical properties of the pristine and carbonized aerogels were studied using the following methods: Raman spectroscopy, X-ray diffractometry, IR spectroscopy, TG/DSC, SEM and TEM. 

The adsorption capacity of the carbonized composite material was studied using the example of Pb(II) extraction from aquatic media. The conducted adsorption experiments showed that the maximum Pb(II) adsorption capacity of this material for the target pollutant was 185 mg/g (reached at pH 6.0), and the contact time was 15 min. The adsorption kinetics were analyzed using pseudo-first-order, pseudo-second-order, and Elovich kinetic models, as well as Weber–Morris and Boyd diffusion models. 

The results of the desorption studies showed a very low desorption rate (0.3% of the pollutant adsorbed) at pH 6.5 and a rate of about 40% (of the pollutant adsorbed) in a strongly acidic medium.

Based on the diffusion models, it can be assumed that the process was complex, combining boundary layer and intraparticle diffusion, from which it can be assumed that the adsorption on the developed graphene-polymer composite proceeded in a mixed diffusion mode.

## Figures and Tables

**Table 1:** Porous space parameters of the nanocomposites.

Material	*S*_BET_, m^2^/g	*S*_DFT_, m^2^/g	*V*_DFT_, cm^3^/g	*V*_micro_, cm^3^/g	*V*_meso_, cm^3^/g
Aerogel	289	228	0.316	0.062	0.258
Carbonized aerogel	315	275	0.747	0.215	0.535

*S_BE_*_T_ and *S_DFT_*—specific surface area of the materials calculated according to the Brunauer–Emmett–Teller (BET) and density functional theory (DFT) models, respectively; *V_DFT_*—pore volume determined according to the DFT model; *V_micro_* and *V_meso_*—volume of micro- and mesopores estimated according to the quenched solid DFT (QSDFT) model, respectively.

**Table 2:** Interplanar distances of the pristine and carbonized aerogels.

Material	Interplanar Distance (d) for 2θ Angle, Å			
	at ~19°	at ~25°	at ~42°	at ~78°
Aerogel	4.63	3.49	2.12	1.22
Carbonized aerogel		3.51	2.13	1.22

**Table 3:** Kinetic and diffusion model equations.

Model	Equation (Integral Form)	
Pseudo-first-order	$q_{t}=q_{e}\left(1-exp\left(-k_{1}t\right)\right)$	(2)
Pseudo-second-order	$q_{t}=\frac{k_{2}q_{e}^{2}t}{1+k_{2}q_{e}t}$	(3)
Elovich	$q_{t}=\frac{1}{\beta }ln\left(1+\alpha \beta t\right)$	(4)
Weber–Morris (intraparticle diffusion)	qt=kid·t12+C	(5)
Boyd (external (film) diffusion)	$F=1-\left(\frac{6}{\pi^{2}}\right)exp\left(-Bt\right)$ $B_{t}=0.4977-\ln\left(1-q_{t}/q_{e}\right)$	(6)

**Note:** *q_e_* (mg/g)—the Pb(II) amount adsorbed at equilibrium time (adsorption capacity); *q_t_* (mg/g)—the Pb(II) amount adsorbed at time *t* (adsorption capacity); *k*_1_ (1/min)—pseudo-first-order adsorption constant; *k*_2_ (g/(mg·min))—pseudo-second-order adsorption constant; *α* (g/mg) and *β* (mg/(g min)—Elovich constants; *k_id_* (g/mg/min^1/2^)—intraparticle diffusion constant; *C* (mg/g)—boundary layer thickness; *F* (mg/g)—adsorbate fraction adsorbed at a different time *t*; *B_t_*—the *F* function.

**Table 4:** Parameters of the kinetic and diffusion models describing the Pb(II) adsorption on the carbonized aerogel.

Experiment	Boyd		Weber–Morris			
$q_{e}$	**R^2^**		$k_{id}$	C	**R^2^**	
~185	0.9647		51.86	16.047	0.9808	
	**Pseudo-first-order**			**Pseudo-second-order**		
	$k_{1}$	$q_{e}$	**R^2^**	$k_{2}$	$q_{e}$	**R^2^**
	0.1708	187.56	0.9972	0.0010	213.17	0.9793
	**Elovich**					
	**β**		**α**		**R^2^**	
	0.0241		433		0.9331	

**Table 5:** Comparative characteristics of Pb(II) adsorption on various materials.

Material Type	Experimental Conditions				
	pH	C_e_, mg/L	m, g	t, min	q_e_, mg/g
Oxidized multiwalled carbon nanotubes (oxMWCNTs6h) [38]	5.0	1	5 mg	20	4.8
Lanthanum-based metal organic framework decorated polyaniline (La-MOF@*x*%PANI) composite [39]	6.0	140	1 g/L	1140	185
Zero-valent nickel nanoparticle decorated polyaniline (PANI-NSA@Ni0) composite nanotubes [40]	5.0	100	0.5 g/L	5	170
Compressible metal organic framework nanofibrous reinforced chitosan aerogel (UiO-66-NH_2_/PANNs/CS) [41]	5.0	100	3 mg	240	84
PANI@APTS-magnetic attapulgite composites (PANI@APTS-Fe_3_O_4_/ATP-0.7) [42]	5.0	100	20 mg	15	130
N-doped carbon aerogel (NCA) microspheres [43]	6.0	250	10 mg	20	209
Chitosan-conjugated magnetite (CH-MNP-CA) nanoparticles [44]	6.1	30	0.5 g/L	60	114
Hermo-sensitive surface ion-imprinted polymers based on multiwalled carbon nanotube composites [45]	6.0	100	20 mg	15	83
Chemical modification of activated carbon (AT-MAC) [46]	5.5	250	0.01	100	250
Magnetic oak wood ash/graphene oxide (Ash/GO/Fe_3_O_4_) [47]	6.0	10	1 g/L	60	99.7%
Carbonized r-GO/o-CNT/PANI aerogel (this work)	6.0	100	0.005	15	185

## Data Availability

The data supporting the reported results were generated during the study.
